# Post-COVID-19 vaccine acute hyperactive encephalopathy with dramatic response to methylprednisolone: A case report

**DOI:** 10.1016/j.amsu.2021.102803

**Published:** 2021-09-06

**Authors:** Abdulrahman F. Al-Mashdali, Yaser M. Ata, Nagham Sadik

**Affiliations:** Department of Internal Medicine, Hamad Medical Corporation, Doha, Qatar

**Keywords:** COVID-19, Vaccination, SARS-CoV-2, Encephalopathy, Adverse reaction, Case report

## Abstract

**Background:**

Since introducing the SARS-CoV-2 vaccination, different adverse effects and complications have been linked to the vaccine. Variable neurological complications have been reported after receiving the COVID-19 vaccine, such as acute encephalopathy.

**Case presentation:**

In this report, we describe a 32-year-old previously healthy man who developed acute confusion, memory disturbances, and auditory hallucination within 24 hours from getting his first dose of the COVID-19 Moderna vaccine.EEG showed features of encephalopathy, CSF investigations were nonspecific, and MRI head did not depict any abnormality. He received five days of ceftriaxone and acyclovir without any benefit.

**Discussion:**

Extensive workup for different causes of acute encephalopathy, including autoimmune encephalitis, was negative. Also, Our patient improved dramatically after receiving methylprednisolone, supporting an immune-mediated mechanism behind his acute presentation. Accordingly, we think the COVID-19 vaccine is the only possible cause of our patient presentation, giving the temporal relationship and the absence of other risk factors for encephalopathy.

**Conclusion:**

the clinician should be aware of the possible neurological complications of the different COVID-19 vaccines. Further research is needed to clarify the pathophysiology of such complications.

## Highlights

• Neurological complications have been reported in patients who received the COVID-19 vaccine.
• It is still not confirmed if these complications are accidentally or casually related to the COVID-19 vaccine.
• The pathophysiology of those complications is still not established, most likely related to immune mediated mechanism.
• Steroid therapy seems to be effective in the treatment of acute encephalopathy related to COVID-19 vaccination.

## 1 Introduction

Severe acute respiratory syndrome coronavirus 2 (SARS‐CoV‐2) appeared in December 2019 and caused the coronavirus disease 2019 (COVID‐19). COVID -19 is a systemic disease condition that can lead to various systemic complications affecting almost any system in the body. Recently, different types of COVID-19 vaccines have been distributed worldwide; however, despite the updated studies on these vaccines and their safety, there are several registered complications that can be related to the vaccine [1]. Neurological complications, such as autoimmune encephalitis, demyelination diseases, Guillain‐Barré syndrome (GBS), seizures, and acute encephalopathy, have been reported in patients who received the COVID-19 vaccine. Nevertheless, it is still speculative if these complications are accidentally or casually related to the COVID-19 vaccine. The pathophysiology of those complications is still not well understood and only based on hypotheses [[2], [3], [4]] [[2], [3], [4]] [[2], [3], [4]]. Herein, we report a case of acute encephalopathy within a day of receiving the first dose of the COVID-19 vaccine (Moderna). Extensive workup, including brain imaging and autoimmune disorders investigation, resulted in negative. Notably, our patient responded dramatically to methylprednisolone that might point to an immune-related phenomenon. Accordingly, we believe that the vaccine is the only possible culprit for this acute presentation in our patient. This case report has been reported in line with the SCARE Criteria [5].

## 2 Presentation of case

A 32-year-old Asian male was brought to the emergency department by his neighbors on May 28th, 2021, because he was seen roaming around his apartment with confusion and agitation. Proper history could not be taken as he was disoriented and amnesic. However, he had no significant past medical history based on his electronic medical record and family information. Also, there was no history of psychiatric illnesses, alcohol use disorder, or other substances abuse. Interestingly, he received the first dose of SARS-Cov-2 vaccination (Moderna vaccine) on May 26th, 2021 (two days before the presentation), and that was the last time seen fine. Upon presentation, he was afebrile and vitally stable. The neurological examination was unremarkable, apart from agitation, disorientation to time, place, person, and memory disturbances.

**Fig. 1:**
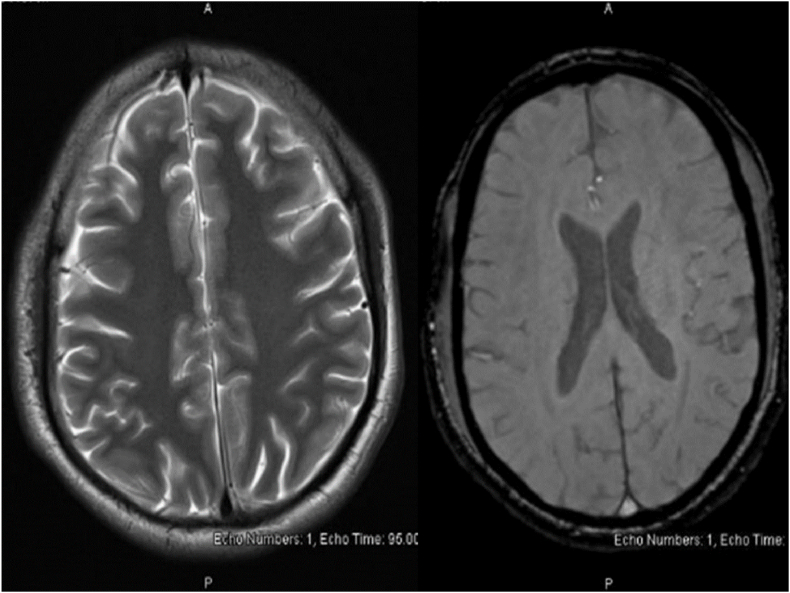
MRI of the brain revealed no abnormality.

His laboratory results, including alcohol and toxicology screen, were negative. COVID-19 PCR test was negative (repeated twice). Lumbar puncture with Cerebrospinal fluid (CSF) studies was done, and it showed elevated protein levels (0.76 gm/L, reference range = 0.15–0.45) with average cell counts (white blood cells of 3 u/L) and glucose levels. Also, CSF oligoclonal band, HSV PCR, VDRL came negative.EEG showed findings suggestive of acute encephalopathy (slowed background activity). Magnetic resonance imaging (MRI) of the brain did not reveal any acute or chronic abnormality (Fig. 1).

During his hospital stay, he received a five-day course of ceftriaxone and acyclovir for suspected meningoencephalitis, but his disorientation and aggressiveness got worse. Additionally, he developed auditory hallucinations and abnormal behaviours. He received multiple doses of haloperidol and lorazepam and finally required bed restraints. Based on his clinical manifestations, autoimmune encephalitis was suspected, and empirical intravenous methylprednisolone (1 g/day) was started on day six of the hospital stay. After two doses of methylprednisolone, he showed a dramatic improvement and became able to understand and answer our questions. He confirmed that he started to develop forgetfulness and mood disturbance within 24 hours of receiving the covid-19 vaccine dose, but he did not remember what happened after that. CSF extensive workup for autoimmune encephalitis (including *anti*-aquaporin-4, anti-myelin basic protein, anti-myelin oligodendrocyte glycoprotein, anti-glial fibrillary acidic protein, *anti*-NMDAR, anti-GAD, and other autoimmune encephalitis antibodies) was negative. He was discharged from the hospital after receiving three days of methylprednisolone. At follow-up in our outpatient clinic (one month after the discharge), he was asymptomatic and fully oriented.

**Patient perspective**: “Actually, this was the first time during my life I felt like I could not remember anything. It was a very terrifying period and I hope that it will not happen again. Obviously, I think that the vaccine is the only possible explanation for my disease. Honestly, I am not sure if I will be able to take the second dose of the vaccine”.

## 3 Discussion

COVID‐19‐related encephalopathy has been reported infrequently since the beginning of the pandemic. Initially, this condition was attributed to direct invasion of the brain by SARS‐CoV‐2; however, SARS‐CoV‐2 RNA usually has not been detected in the cerebrospinal fluid (CSF) of those patients with encephalopathy. In addition to that, CSF analysis, neuroimaging, histopathological findings, and the clinical response to immunosuppressant therapy (such as a steroid), found to be consistent with immune‐related pathogenesis involving the brain rather than a direct infectious process targeting the brain. Thus, a cytokine‐mediated inflammatory process is proposed as the key pathophysiological mechanism for COVID‐19‐related encephalopathy, known as cytokine storm-associated encephalopathy (CySE) [[6], [7], [8]].

Neurological symptoms like pain, headache, dizziness, or muscle spasms are common and self-limited adverse effects after receiving the COVID-19 vaccine. However, major neurological complications, despite the unproven causality, have been reported since the introduction of the COVID-19 vaccine, such as facial palsy, Guillain-Barre syndrome(GBS), seizures, strokes, transverse myelitis, and acute disseminated encephalomyelitis (ADEM) [2].In addition, an unexplained acute encephalopathy state has also been described after receiving the COVID-19 vaccine [3,9]. Regarding the pathogenesis of COVD-19 vaccine-associated complications, It has been proposed that spike protein expression by human cells (after translation of the mRNA COVID-19 vaccine by human cells) might trigger an inflammatory reaction, similar to that induced by the virus itself, leading to those neurological complications [3,6,10]. However, elevated inflammatory markers, including cytokines, were not detected in many reported cases of covid-19 vaccine-related encephalopathy (based on USA's Vaccine Adverse Event Reporting System) [9]; Therefore, we believe that other unknown mechanisms might lead to such complications.

Our patient developed this acute hyperactive encephalopathy (based on clinical presentation, EEG findings, and MRI) within 24 hours of vaccine administration. Initially, meningoencephalitis was suspected; however, despite receiving five days course of ceftriaxone (to cover bacterial meningitis) and parenteral acyclovir (to cover herpes encephalitis), his condition had deteriorated more. We extensively investigated the patient for different causes of an acute encephalopathy, including autoimmune encephalitis work up, without reaching any diagnosis. Also, the patient's dramatic response to methylprednisolone might point to an immune-mediated mechanism behind his condition. Our case nearly resembles a recently reported case by L. Baldelli et al. [8] that describes the occurrence of hyperactive acute encephalopathy post-COVID-19 vaccine. Accordingly, we can link the development of acute encephalopathy in our patient to the vaccine due to the temporal relationship and the absence of other risk factors for encephalopathy in our patient.

## 4 Conclusion

Up to the best of our knowledge, this is the first reported case presented with acute encephalopathy after receiving the COVID-19 vaccine in Qatar. Our patient markedly improved with corticosteroid therapy supporting immune-mediated pathogenesis behind his clinical presentation. Further studies are required to describe the pathogenesis of COVID-19 vaccination-related complications and their optimal management. Finally, we believe that the vaccine's benefits outweigh the risk of any possible complications associated with its use. Albeit, clinicians should be aware of the COVID-19 vaccine's possible complications, and if they happen, they should not be considered coincident events and should be reported as soon as possible.

## Ethical approval

Case report was approved by Hamad Medical Corporation Medical Research Centre.

## Sources of funding

Open access funding was provided by Qatar National Library (QNL)

## Author contribution

AFA: patient care, data gathering, literature review, manuscript writing. YMA: patient care, manuscript writing. NS: editing and revision of the final manuscript. All authors reviewed, edited, and approved the final version of the manuscript.

## Registration of research studies

N/A.

## Guarantor

Abdulrahman F. Al-Mashdali.

## Consent

Written informed consent was obtained from the patient for publication of this case report and the accompanying image. A copy of the written consent is available for review by the Editor-in-Chief upon request.

## Consent for publication

Written informed consent was obtained from the patient for publication of this case report and the accompanying image. A copy of the written consent is available for review by the Editor-in-Chief upon request.

## Availability of data and materials

The datasets used and/or analyzed during the current study are available from the corresponding author on reasonable request.

## Provenance and peer review

Not commissioned, externally peer reviewed.

## Declaration of competing interest

The authors have no competing of interest to declare.

## Declaration of competing interest

All authors have no conflict of interest to declare.
